# An investigation of the prevalence and diversity of *Anisakis* in China: marine food safety implications

**DOI:** 10.3389/fmicb.2024.1399466

**Published:** 2024-05-17

**Authors:** Min-hao Zeng, Chuan-tao Fang, Xiao-xu Wang, Abdul Qadeer, Yang-yuan Qiu, Xiao-mei Hong, Mohamed H. Mahmoud

**Affiliations:** ^1^School of Biotechnology, Jiangsu University of Science and Technology, Zhenjiang, China; ^2^Shanghai Tenth People's Hospital, Shanghai, China; ^3^Department of Cell Biology, School of Life Sciences, Central South University, Changsha, China; ^4^State Key Laboratory for Diagnosis and Treatment of Severe Zoonotic Infectious Diseases, Key Laboratory for Zoonosis Research of the Ministry of Education, Institute of Zoonosis, College of Veterinary Medicine, Jilin University, Changchun, China; ^5^School of Public Health, Shantou University, Shantou, China; ^6^Department of Biochemistry, College of Science, King Saud University, Riyadh, Saudi Arabia

**Keywords:** *Anisakis*, fish, China, prevalence, meta-analysis

## Abstract

*Anisakis* can cause Anisakiasis in humans if raw or undercooked fish is consumed. Symptoms of infection may include vomiting, acute abdominal symptoms, or allergies. In this study, we collected 187 commercially available marine fish from the Yellow Sea, East China Sea, and South China Sea. Among them, 79 were found positive containing 520 *Anisakis* worms. The average prevalence rate was found 42% in this investigation. Ninety-two worms from different sea areas were selected and analyzed for identification, revealing the presence of five different species, which are *Anisakis pegreffii*, *Hysterothylacium aduncum*, *Hysterothylacium zhoushanense*, *Hysterothylacium amoyense*, and *Hysterothylacium* sp. In the meta-analysis, three databases: PubMed, CNKI, and BaiduXueshu were searched for surveys on the prevalence of *Anisakis* in Chinese waters from January 2000 to December 2023. A total of 26 studies were included in this analysis of which 25 publications were retrieved from different databases and one being the present study. The pooled prevalence of *Anisakis* was 45% among commercially available marine fish. Variances in the prevalence of *Anisakis* were noted among the four seas, with the highest rates in the East China Sea and the Bohai Sea, reaching 53% [0.38; 0.68] and 49% [0.36; 0.62], respectively. The Prevalence of *Anisakis* infection was significantly higher in astern parts such as Liaoning, Shanghai, and Zhejiang. Analysis of the host fish subgroups revealed that the orders of Anguilliformes, Scombriformes, and Gadiformes had high rates of infection. These findings suggest a significant prevalence of *Anisakis*, posing an increasing risk of infection for individuals. This study provides impactful information for implementing preventative measures against *Anisakis*.

## 1 Introduction

Food safety is a significant concern for human health, with foodborne parasites being a major risk factor (Zhao et al., 2024). The family of Anisakidae, which includes the *Anisakis* genus, is a parasitic nematode commonly found in marine fish. Only *Anisakis simplex* and *Anisakis pegreffii* have been reported to cause human Anisakiasis (Cipriani et al., 2021). This issue has emerged as a significant health concern globally, particularly in countries or regions like Japan, Korea, China, Taiwan, Portugal, Chile, and the places where people have a culture of consuming raw or lightly processed seafood (Mattiucci et al., 2018). For instance, in Japan in 2021, 48% of reported food poisoning cases (344/717) were attributed to *Anisakis* larvae (Kodo et al., 2023). A recent study examining ready-to-eat mackerel (*Scomber japonicus*) samples in Japan found that 54% (244/448) were infected with *Anisakis* nematode (Ohnishi et al., 2023). In Chile, a survey indicated that almost all Chilean hake (*Merluccius gayi*) and sea bream (*Brama australis*) were parasitized with *Anisakis* (Muñoz-Caro et al., 2022). Another survey in Portugal on European hake (*M. merluccius*) showed that *Anisakis* had a prevalence of 76.7% (Santos et al., 2022). In the East China Sea, the infection prevalence of Anisakidae larvae was as high as 95.1% (116/122) (Kong et al., 2015).

Humans can become infected with Anisakiasis by consuming raw or undercooked fish that contain *Anisakis*. This can lead to serious symptoms including severe abdominal pain, nausea, vomiting, and other conditions like peptic ulcers, appendicitis, or peritonitis, and the most worrying manifestation is allergic reactions ranging from hives to anaphylaxis (Villazanakretzer et al., 2016). However, many Chinese people lack an in-depth understanding of the risks associated with consuming raw or undercooked marine fish (Qin et al., 2013; Chen et al., 2018). These findings highlight a significant risk of Anisakiasis becoming an epidemic in China as well as globally. The main aim of our study was to gain a more comprehensive understanding of the prevalence of *Anisakis* in China, we conducted a study by collecting marine fish samples from various Chinese waters to investigate the prevalence of *Anisakis* infections and to construct a haplotype network to study the relationship among *Anisakis* from different regions. Additionally, we performed a systematic review and meta-analysis of relevant studies since 2000.

## 2 Materials and methods

### 2.1 Fish and parasite collection

A total of 187 marine fish, from eight orders, were collected from the Yellow, East, and South China Seas of China from August to October 2023. The abdominal cavity, digestive tract, and reproductive glands of the fish were carefully observed under white light, and the flesh of the fish was carefully scraped and checked for the presence of worms in the muscle tissue. The fish tissue adhering to the worms’ surface was removed, and the worms were washed three times with saline solution. Live worms were kept in saline before proceeding to the next step in the study.

### 2.2 Identification of worms

The nematodes were stained with carmine and examined under a light microscope to analyze their morphology for preliminary identification. A total of 92 nematodes from various waters were randomly selected for molecular characterization. Genomic DNA was extracted from the nematode using SDS lysis buffer (Tris–HCl 100 mM, BIOFROXX, Germany; EDTA 25 mM, Diamond, China; NaCl 500 mM, Sangon, China) and proteinase K (Qiagen, Germany). DNA amplification based on the ITS1 sequence was performed using ITS1-F (5′-AAA GTC GTA ACA AGG TTT CCG TAG −3′) and ITS1-R (5′-GAA CCG AGT GAT CCA CCG CCA A − 3′) primers (which were designed based on GenBank accession: MT820020.1) in a SimpliAmp Cycler (Thermo Fisher Scientific, United States) with the following thermal cycling conditions: initial denaturation 94°C for 5 min, followed by 39 cycles denaturation at 94°C for 30 s, annealing at 51.7°C for 30 s, and extension at 72°C for 30 s, with the final extension at 72°C for 5 min. The PCR reaction mixture contained 10 μL of 2 × Hieff PCR Master Mix (Yeasen, China), 0.4 μL ITS1(10 μM) forward and reverse primer each, 1 μL of template genomic DNA, and ddH2O up to 20 μL. The PCR products were sent to Tsingke (Guangzhou) Technology Company for Sanger sequencing. The obtained sequences were analyzed, edited and trimmed by Clustal W in MEGA v5.2.2 (Tamura et al., 2011). The phylogenetic relationship tree was reconstructed using the Maximum Parsimony method (bootstrap = 1,000) based on the ITS1 sequences of the samples and related species by MEGA (v 5.2.2).

### 2.3 Study selection and data extraction

PubMed,^1^ the Chinese National Knowledge Infrastructure (CNKI database, https://www.cnki.net/), and Baidu Xueshu (Search engine, https://xueshu.baidu.com/) were used for literature searches on December 13th, 2023. The search terms “*Anisakis* & epidemiological” and “*Anisakis* & surveys” were employed for Chinese articles search, while “*Anisakis* & China” was used for English articles search. The inclusion criteria for literature were as follows: (1) articles published after 2000; (2) Epidemiological research studies; (3) focusing on *Anisakis* nematodes; (4) Samples collected from Chinese waters. Review and conference papers were excluded to avoid duplication. Subsequently, the included articles’ full text was thoroughly checked and excluded by the following criteria: (1) Not being an epidemiological survey; (2) Duplicative data; (3) Fish samples ≤50; (4) ambiguous sample collection time or location; (5) Insufficient information of fish samples to identify order level; (6) Lack of nematode identification; (7) focusing on marine mammals hosts such as dolphins; (8) unavailability of full text. Data from the included papers were extracted into summary tables. The information was recorded in the following way: first author, publication year, sampling year, geographical region, sample size, infection size, and identification of fish species at the order level. Quantitative analyses were performed using the “Meta” package (v 4.11–0) in the R Studio software (v 1.2.5019). Forest plots displayed the combined estimates with 95% confidence intervals (CI), while heterogeneity was evaluated using Q and I^2^ statistics. Potential publication bias was assessed via Funnel plots and Egger regression tests, focusing on factors such as sampling years, sampling location, and fish orders. Prevalence maps were created using the “ggmap” package. For the subgroup of fish orders, only those with 4 or more supporting studies were included for analysis.

## 3 Results

### 3.1 Study selection and data extraction

**Figure 1:**
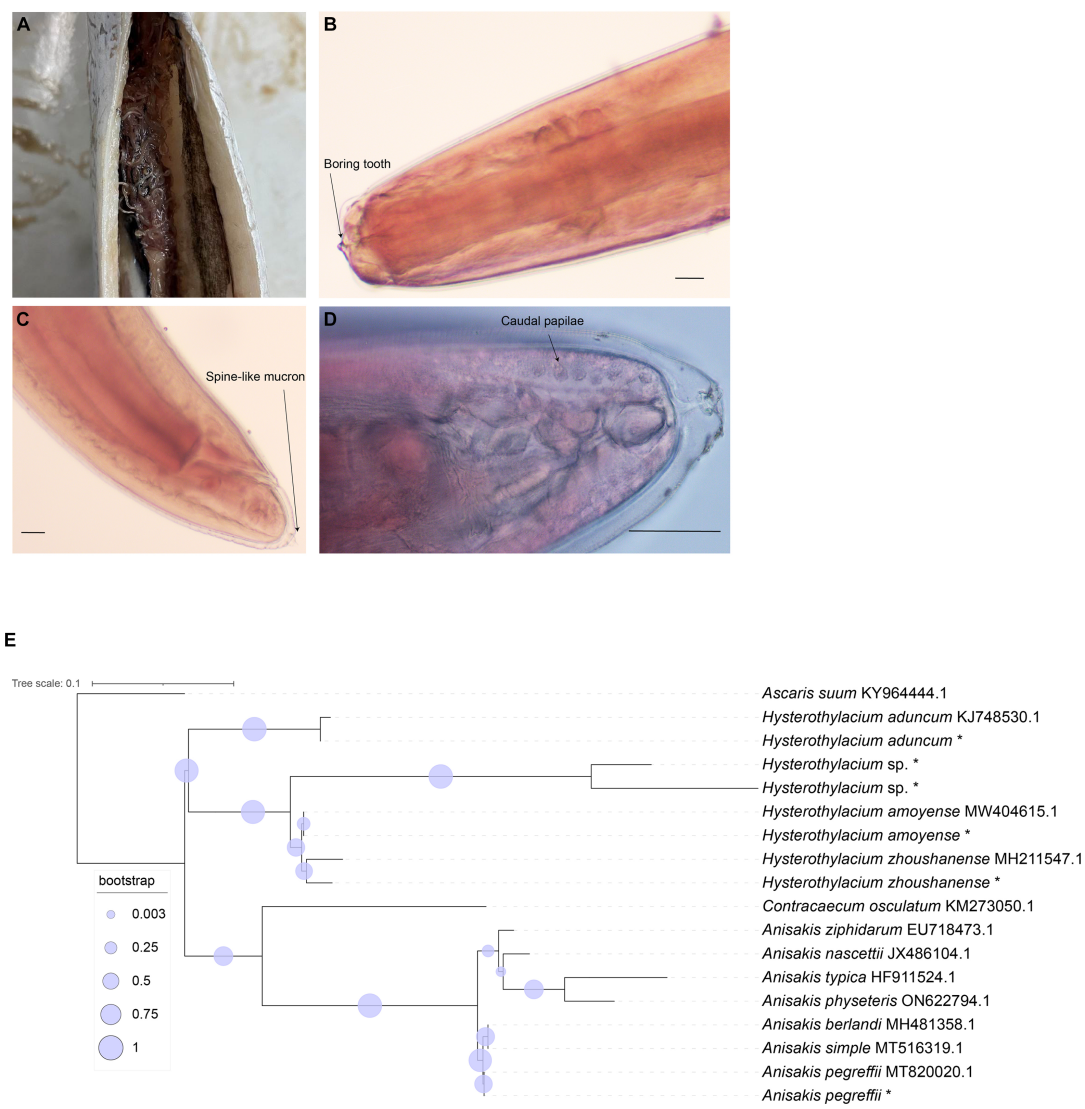
Morphological identification of collected nematode and phylogenetic relationship tree reconstructed based on ITS1 sequences. **(A)** Nematodes in the abdominal cavity of fish. **(B)** The anterior end of the nematodes (carmine staining, ×100). **(C)** The posterior end of the nematodes (carmine staining, ×100). **(D)** The posterior end of the nematodes (carmine staining, ×400). Bar: 100 μm. **(E)** Phylogenetic relationship tree by ITS1 sequences, * means the samples in this study.

In this study, a total of 187 marine fish samples were collected, of which 79 were found to be infected with *Anisakis*, resulting in the isolation of 520 worms (Figure 1A). The average infection rate was found approximately 42%, as shown in Table 1. The isolated nematodes were characterized as white and measured 1–2 cm in length. After staining and examination under a microscope, we found that the larvae lack the complex structure seen in the adults. In larvae only on the anterior end, a tilted boring tooth was observed (Figure 1B). And the larvae were characterized by a short (tail length, 0.1–0.15 mm), conical, and short, blunt tail with a spine-like mucron (Figure 1C). Although the arrangement of papillae (about 10 μm in diameter) at the caudal end was clearer, but it was still hard to reliably determine the species and type to which they belong (Figure 1D) (Quiazon et al., 2009; Mattiucci et al., 2018).

**Table 1:** *Anisakis* detected in various fish samples from different Chinese sea areas.

Regions	Fish orders	No. of fishes	No. of infection
Yellow Sea	Perciformes	28	23
	Mackerels	5	1
East China Sea	Perciformes	29	29
	Aulopiformes	23	0
South China Sea	Mugiliformes	14	0
	Pleuronectiformes	11	2
	Tetraodontiformes	5	2
	Clupeiformes	8	3
	Beloniformes	4	0
	Aulopiformes	5	2
	Perciformes	42	12
	Mackerels	13	5
Total		187	79

With *Ascaris suum* as the outgroup, the MP tree topology as inferred from the phylogenetic analysis of the sequences obtained at the ITS1 region of rDNA of nematodes showed that Anisakidae and Raphidascaridae clustering in two clades with high probability value (70, 97%), respectively. The classification of *Hysterothylacium* spp. were very clear, and all of them have greater than 50% support probability value. The *Hysterothylacium* sp. isolated in this study was more closely related to *H. amoyense* and *H. zhoushanense*, and *H. aduncum* was more distantly related to other *Hysterothylacium* spp. And *H. amoyense*, *H. zhoushanense*, *H. aduncum*, isolated in this study all formed sister strains with the corresponding reference sequences. In the other clades clustered by Anisakidae, the genus *Anisakis* spp. were distinguished from *Contracaecum osculatum* (70% probability value). In clades of *Anisakis* spp., *A. berlandi*, *A. simplex* and *A. pegreffii* were distinguished by forming a small clades with high probability value. The *A. pegreffii* isolated in this study formed sister strain with *A. pegreffii* ITS1 reference sequence. The results of molecular identification were presented in Table 2, with the phylogenetic relationships illustrated in Figure 1E.

**Table 2:** Molecular identification of nematodes from different regions.

Regions	Number of identified specifies					
	*A. pegreffii*	*H. aduncum*	*Hysterothylacium.* sp.	*H. zhoushanense*	*H. amoyense*	Total
Yellow Sea	12	6	16	–	–	34
East China Sea	16	3	2	1	–	22
South China Sea	8	–	12	4	12	36
Total	36	9	30	5	12	92

Among 92 nematodes analyzed, 36 were identified as *A. pegreffii*, 9 from *H. aduncum*, 5 from *H. zhoushanense*, 12 from *H. amoyense*, and 30 from *Hysterothylacium* sp., *A. pegreffii* and *Hysterothylacium* sp. were found across all three sea samples, while *H. aduncum* was not detected in the South China Sea samples and *H. zhoushanense* was not detected in the Yellow Sea samples. Additionally, *H. amoyense* was only detected in the South China Sea samples.

### 3.2 META analysis

**Figure 2:**
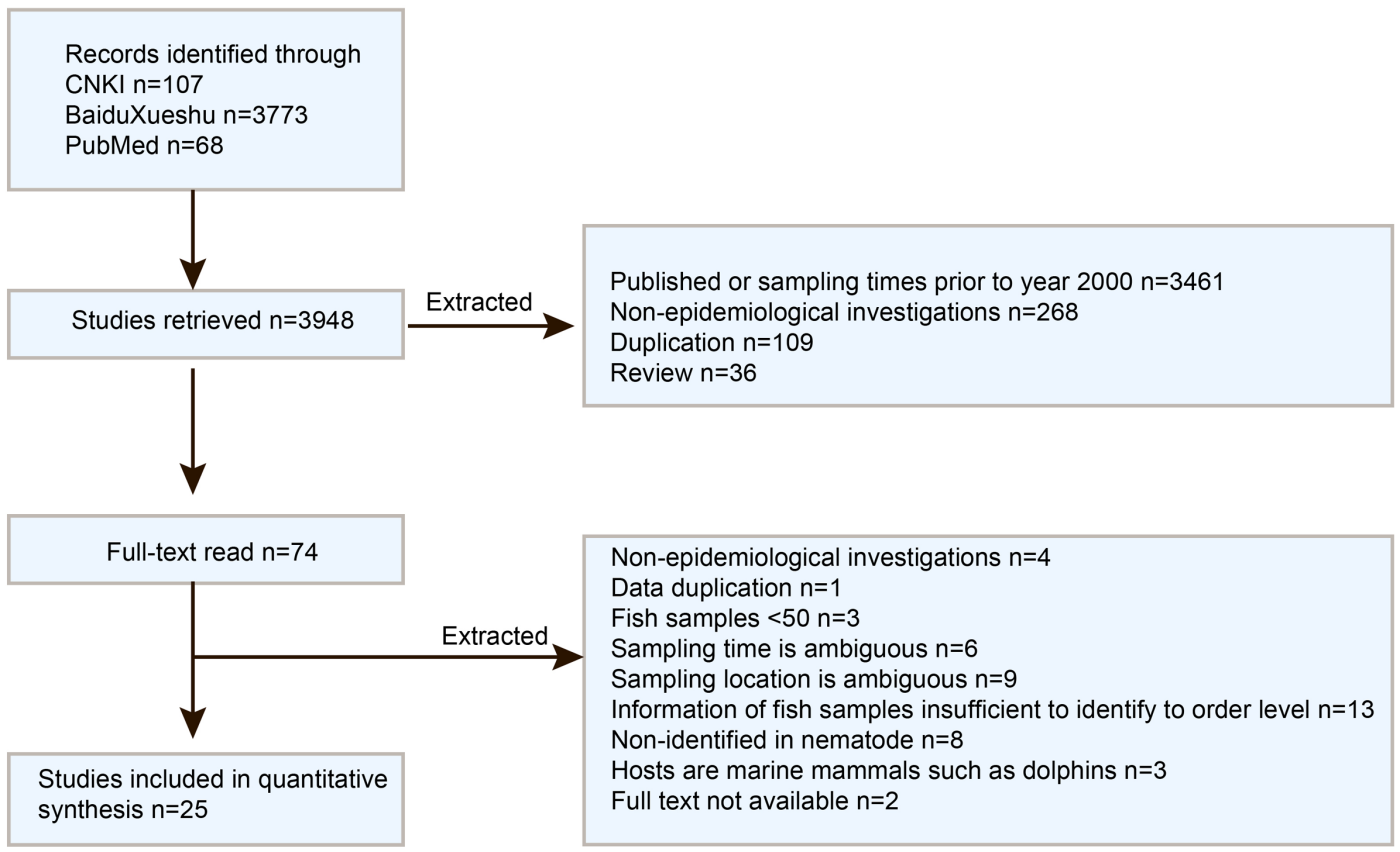
Flowchart of article Screening for analysis.

A total of 3,880 publications were retrieved from the three databases and search engines, with 25 full-text studies following the inclusion criteria (see Figure 2). These studies covered the locations in China, including the Bohai, Yellow, East, and South China Seas (Supplementary Table S1). The meta-analysis comprised a total of 26 studies, which consist of 25 previously published articles and one from our ongoing research.

**Figure 3:**
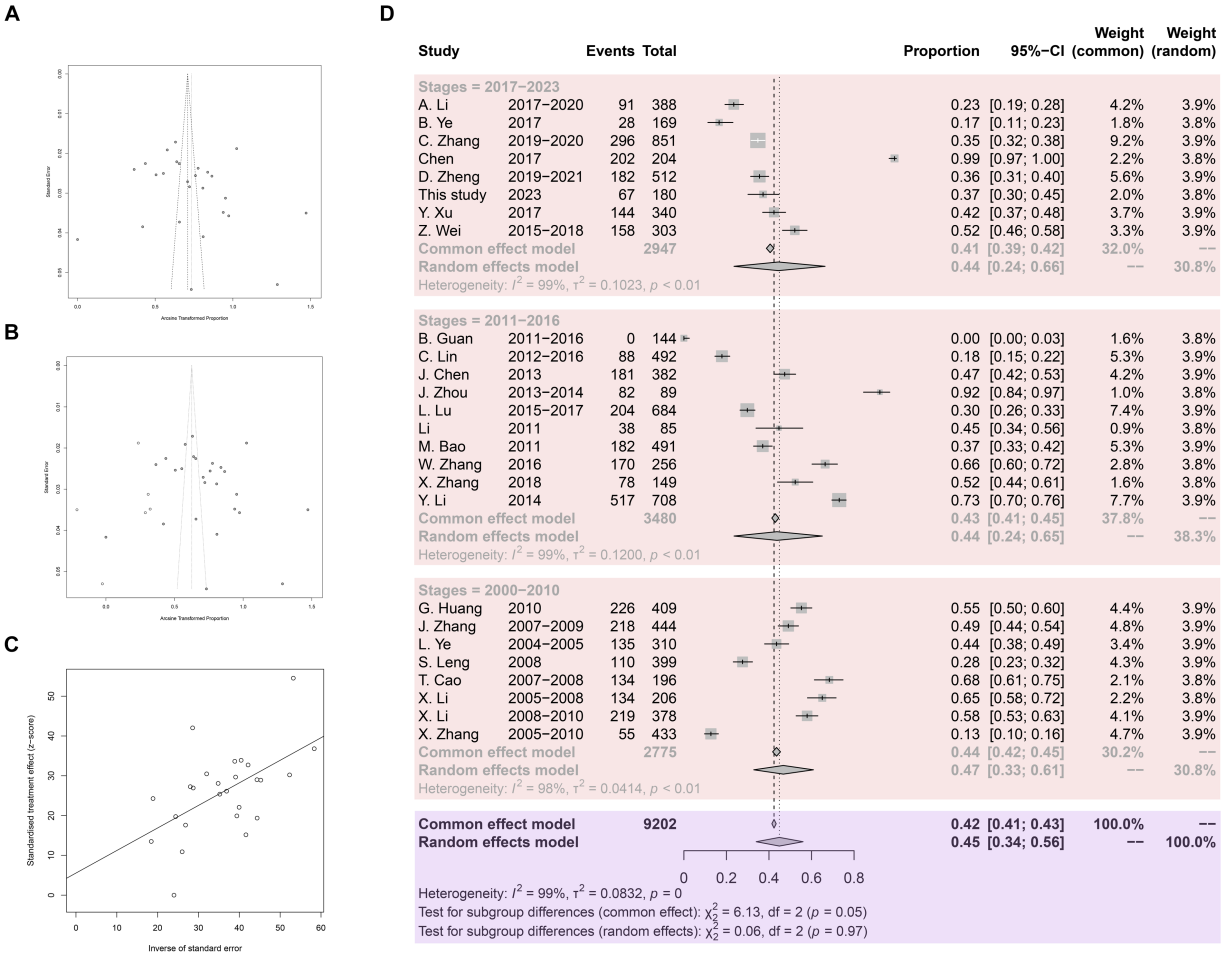
Analysis for the examination of publication bias. **(A)** Funnel plot with pseudo 95% confidence interval limits. **(B)** Funnel plot with trim and filling analysis. **(C)** Egger’s test for publication bias. **(D)** Forest plot of *Anisakis* prevalence in China.

Meta-bias analysis indicated no significant publication bias in the included studies. Both the funnel plot and the trim and fill funnel plot exhibited more symmetry (Figures 3A,B). Egger’s test yielded a *p*-value of 0.4062, with a bias value of 5.5546 (SE = 6.5700) (Figure 3C). Sensitivity tests demonstrated that omitting any study did not significantly affect the combined overall prevalence (Supplementary Figure S1). Hence the meta-analysis results were deemed reliable.

A total of 9,202 fish samples were included, with 3,939 infected with *Anisakis*, resulting in an arithmetic prevalence of 42.8%. Heterogeneity indices are displayed using a forest plot. Random-effect and common-effect models were used to estimate the prevalence of each subgroup (Figure 3D). The heterogeneity I^2^ value for all studies was above 98%, greater than 50%, so the random-effect model results were used. The pooled prevalence of *Anisakis* infection in fish in China since 2000 was 45%. Subgroup analysis by time revealed a pooled prevalence of 47% in 2000.1–2010.12, 44% in 2011.1–2016.12, and 44% in 2017.1–2023.12.

**Figure 4:**
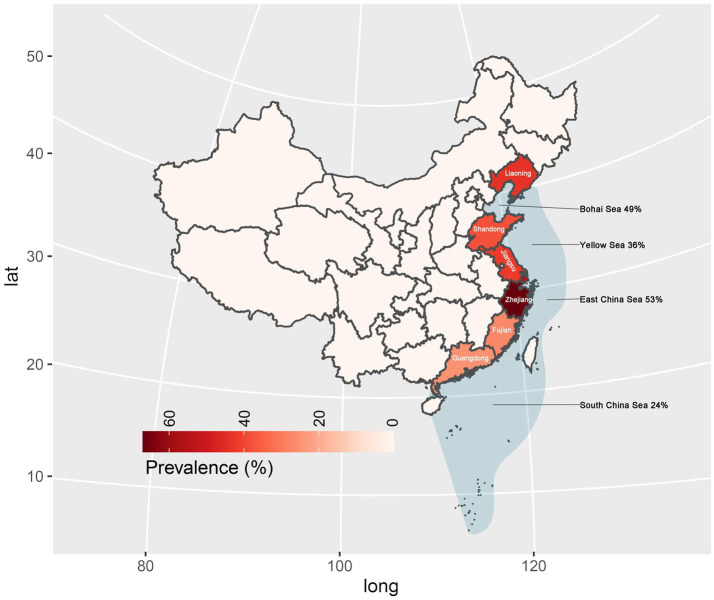
Prevalence of *Anisakis* in marketed marine fish in China.

In subgroup analysis by provinces and cities, Zhejiang province exhibited the highest prevalence at 67%, supported by 10 studies with a total sample size of 2,659 and 1702 positive counts. Conversely, Guangdong Province had the lowest prevalence at 25%, supported by 5 studies with a total sample size of 823 as shown in Figure 4. Sea regions as subgroups showed a prevalence higher than 50% in the East China Sea, 49% in the Bohai Sea, only 36% in the Yellow Sea, and only 24% in the South China Sea as mentioned in Figure 4. When we analyzed different host fish order as a subgroup, the highest prevalence was observed in the order Gadiformes at 86% based on only 4 studies with a total sample size of 42 out of which 35 positive counts. Lophiiformes followed closely with a prevalence of 77%, supported by 8 studies with a total sample size of 154, of which 79 were positive. The highest number of supporting studies and samples was for Perciformes, with a prevalence of 49% from 24 studies with a total sample size of 5,059, and 2,305 positives. The random effect of Scombriformes and Anguilliformes was 61 and 74% respectively, supported by 22 and 11 studies. Conversely, there were no positives in the order of Sepioidea, supported by 9 studies with 186 samples. The prevalence was also low in the orders Teuthida and Beloniformes, at 5 and 6%, respectively, as detailed in Table 3.

**Table 3:** Pooled prevalence of *Anisakis* in China.

Subgroups			No.		Random-effect prevalences (95% CI)
		Studies	Samples	Positive	
Fish order	Scorpaeniformes	8	196	115	32% [0.03; 0.74]
	Teuthida	11	208	29	5% [0.00; 0.17]
	Anguilliformes	11	422	379	74% [0.44; 0.95]
	Aulopiformes	8	193	51	35% [0.08; 0.69]
	Clupeiformes	13	329	39	14% [0.04; 0.29]
	Pleuronectiformes	14	359	101	10% [0.02; 0.23]
	Scombriformes	22	1,114	640	61% [0.46; 0.75]
	Beloniformes	9	84	9	6% [0.00; 0.29]
	Gadiformes	4	42	35	86% [0.17; 1.00]
	Perciformes	24	5,059	2,305	49% [0.37; 0.61]
	Rajiformes	4	80	24	40% [0.01; 0.90]
	Lophiiformes	8	154	79	77% [0.33; 1.00]
	Sepiida	9	186	0	0%[0.00; 0.01]
Sea region	Bohai Sea	4	1,422	662	49% [0.36; 0.62]
	Yellow Sea	7	1,306	430	36% [0.24; 0.50]
	East China Sea	15	5,223	2,370	53% [0.38; 0.68]
	South China Sea	5	1,251	477	24% [0.05; 0.51]
Province/City	Liaoning	3	1,041	444	44% [0.35; 0.52]
	Shandong	4	752	305	38% [0.18; 0.61]
	Jiangsu	4	910	334	42% [0.28; 0.61]
	Shanghai	3	1,134	276	54% [0.01; 1.00]
	Zhejiang	10	2,659	1702	67% [0.51; 0.80]
	Fujian	4	1,534	418	27% [0.20; 0.35]
	Guangdong	5	823	312	25% [0.03; 0.59]

## 4 Discussion

The present study investigated the prevalence of infection in positive samples of *Anisakis*, which was found to be 42%. Among the genus *Anisakis*, only one species *A. pegreffii* was detected, accounting for 39.13% (36/92) of all samples. Within the genus *Hysterothylacium*, four species were detected, three of which were validated and one unspecified, representing 6.87% (56/92) of all samples. Among these, *H. aduncum* accounted for 16.07% (9/56) of the genus *Hysterothylacium*, *H. zhoushanense* for 8.93% (5/56), *H. amoyense* for 21.43% (12/56), and *Hysterothylacium* sp. for 53.57% (30/56). Furthermore, various species exhibit distinct geographical distributions. For instance, all 12 samples collected from the South China Sea were identified as *H. amoyense*. *H. zhoushanense* was absent in the Yellow Sea and only 6 out of 22 samples of the genus *Hysterothylacium* were detected in the East China Sea, with the remaining 16 being *A. pegreffii*. The distribution of *A. pegreffii* is more evenly across the various seas. During the survey of *Nemipterus japonicus* in the South China Sea, five species of nematodes belonging to the genus *Hysterothylacium* were identified. Among these, *H. amoyense* had the highest prevalence, consistent with the findings of the present study. Although *Hysterothylacium* sp. was also detected in 12 samples, *H. amoyense* was exclusively found in the South China Sea (Guo et al., 2020). In a survey of 42 monkfish in the East China Sea, the overall prevalence of infection was 100%, with 83.3% of *A. pegreffii* infections as the predominant species (Zhang et al., 2018). Similarly, in another survey of marine fish samples from the East China Sea and the Pacific coast of central Japan, *A. pegreffii* was the predominant species, accounting for 84.8% of all larvae examined (Kong et al., 2015). In this study, *A. pegreffii* was also the predominant species in the East China Sea. Consistency with previous research varies among the samples collected from the Yellow Sea. This study identified a greater number of *Hysterothylacium* compared to *Anisakis*, six were identified as *H. aduncum* while the remaining 16 were *Hysterothylacium* sp. In contrast, a survey conducted in 2007 on 123 fish in the Yellow Sea revealed that 197 out of 200 nematodes collected belonged to the *Anisakis* genus, with the remaining three being of the *H. aduncum* (Zhang et al., 2007).

This meta-analysis conducted a comprehensive analysis of *Anisakis’* prevalence in the Chinese seas from 2000 to 2023. The finding revealed consistently high prevalence rates across all three stages, ranging from (44–47%). Encouragingly, there seems a decline in the prevalence during the recent period (2011–2023) compared to the earlier stages. However, a slight decrease over a prolonged period may simply be within the equilibrium and not significant enough to warrant optimism. In contrast, China currently produces 60.3 million tons of aquaculture, and according to FAO forecasts, this production is expected to increase 31.1% by 2030 (FAO, 2018; Hao et al., 2020). With such a large production, *Anisakis* is highly prevalent in China’s marine areas (Ding et al., 2022). Therefore, as a result, the overall risk of people becoming infected with *Anisakis* is greatly increasing.

Subgroup analyses based on sampling provinces indicated an exceptionally high prevalence of *Anisakis* in marine fish within the eastern coastal provinces or cities of China (25–67%). Conversely, the prevalence was notably lower in the southern coastal provinces and marine fish in northeastern China. Further subgroup analyses based on sampled sea area, mirrored the province-based findings. *Anisakis* exhibited the highest prevalence rates in the Yellow Sea (36%) and the East China Sea (53%), while showing relatively lower prevalence rates in the Bohai Sea (49%) and the South China Sea (24%). In China, Guangdong province is well known for its cultural practice of consuming raw fish. As a fish-borne parasite of concern, the infection rates and prevalence of *Clonorchis sinensis* have been reported to be very high in Guangdong Province (Deng et al., 2020; Huang et al., 2024). However, no case reports of human Anisakiasis have been reported in Guangdong Province (South China Sea). In a study involving asthma patients in Guangzhou, 8.8% (5/57) were found to be allergic to the *Anisakis simplex* component (rAni s 3) (Hu et al., 2019).

In Fujian Province (East China Sea) only one case was reported, however, the prevalence of *Clonorchis sinensis* infection also reached 0.6% (274/45736) (Cheng et al., 2005; Shu-ling et al., 2022). Despite Zhejiang Province (East China Sea) also has a tradition of consuming raw fish, surprisingly did not report any cases even when the prevalence of *Anisakis* in marine fish was as high as 67%. A risk survey in Jiangsu Province (Yellow Sea) showed that 21.6% of the people consumed raw or semi-raw marine fish and only 13.0% had ever heard of *Anisakis* spp. And the seroprevalence of the anti-*Anisakis* IgG antibody was 7.0% in the study (Mao et al., 2020). In a survey of 387 subjects in Shandong Province (Yellow Sea), only 4.9% (19/387) of the population were aware of the dangers of Anisakiasis, and 7% (27/387) preferred to consume raw or semi-raw marine fish. However, with a prevalence rate of 38% of *Anisakis* in marine fish in Shandong Province, no human Anisakiasis infections were detected in hospitals (Dan et al., 2023). Although the first clinical case of *Anisakis* infection in China was reported from Liaoning Province (Bohai Sea) in 2013, no further cases have been reported in Liaoning Province (Qin et al., 2013). However, the prevalence of *Anisakis* in marine fish in Liaoning Province remains as high as 44%.

Cod is a popular and affordable seafood product in China (Xiong et al., 2016). However, the high contamination rate of *Anisakis* in Chinese waters is a concern for both the market and consumers. China produced over 60,000 tons of eels annually, with 39,000 tons of baked eels exported in 2020 (Ting-ting et al., 2019). Eel processing techniques include baking, smoking, frying, salting, and canning (Gómez-Limia et al., 2021). Even though some treatments cook the fish sufficiently, there is still a risk of sensitization because of the heat-resistant allergen from *Anisakis* (Moneo et al., 2005; Kochanowski et al., 2020). The Perciformes and Scombriformes are the main fish as food on the table, additionally, the by-products of Scombriformes including organs, are used in the manufacture of fishmeal and fish oil (Guerard et al., 2002; Bougatef et al., 2010). These marine fish are close to the market and consumers, yet the contamination of *Anisakis* is so serious. But it has not attracted enough attention.

## 5 Conclusion

This study revealed a notable concern regarding the prevalence of *Anisakis* in marine fish in Chinese waters, reaching as high as 45% between 2000 to 2023. Moreover, the study highlighted a growing risk of exposure to *Anisakis* due to the escalating demand for aquatic food in China. This study marks the first comprehensive systematic evaluation and meta-analysis of *Anisakis* prevalence in marine fish, along with its associated risk factors in China. The findings offer impactful insights for the implementation of preventive measures against *Anisakis* infection.

## Data availability statement

The datasets presented in this study can be found in online repositories. The names of the repository/repositories and accession number(s) can be found in the article/Supplementary material.

## Ethics statement

The manuscript presents research on animals that do not require ethical approval for their study.

## Author contributions

M-hZ: Conceptualization, Data curation, Formal analysis, Investigation, Methodology, Resources, Software, Writing – original draft, Writing – review & editing. C-tF: Formal analysis, Methodology, Software, Visualization, Writing – review & editing. X-xW: Conceptualization, Investigation, Resources, Writing – review & editing. AQ: Writing – review & editing. Y-yQ: Writing – review & editing. X-mH: Resources, Writing – review & editing. MM: Writing – review & editing.

## Footnotes

^1^ https://pubmed.ncbi.nlm.nih.gov/
