# Supplementary material for: An investigation of the prevalence and diversity of Anisakis in China: marine food safety implications
Source: Front Microbiol. 2024 May 17;15:1399466. doi: 10.3389/fmicb.2024.1399466 (PMC11140045; doi:10.3389/fmicb.2024.1399466)
Supplement: Supplementary file 1 [file Table_1.DOCX]

Supplementary Material

# Supplementary Data

Supplementary Material should be uploaded separately on submission. Please include any supplementary data, figures and/or tables.

Supplementary material is not typeset so please ensure that all information is clearly presented, the appropriate caption is included in the file and not in the manuscript, and that the style conforms to the rest of the article.

# Supplementary Figures and Tables

For more information on Supplementary Material and for details on the different file types accepted, please see [here](https://www.frontiersin.org/guidelines/author-guidelines#supplementary-material).

## Supplementary Figures

**
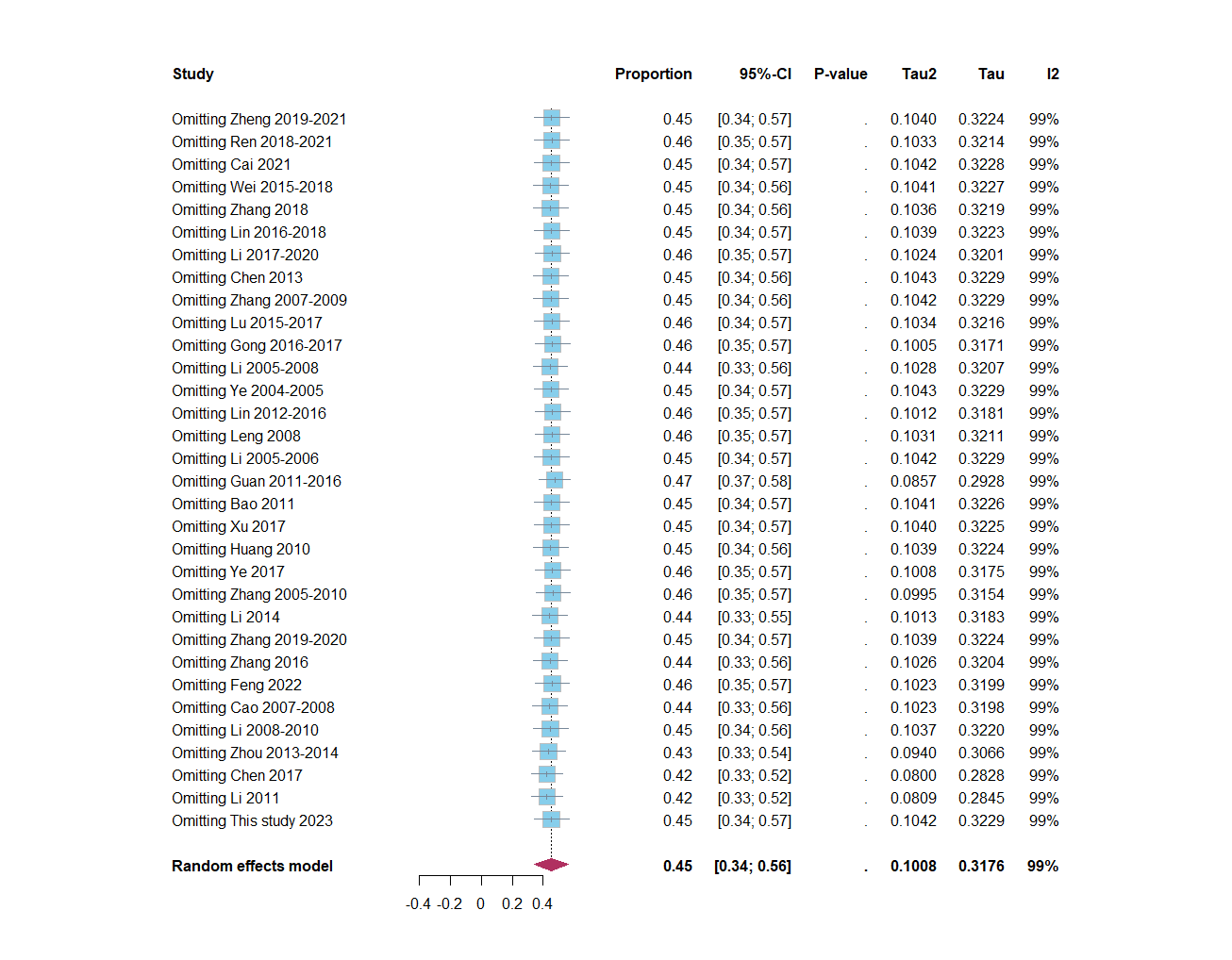
**

**Supplementary Figure S1.** Sensitivity analysis results.

## Supplementary Tables

**Supplementary Table S1.** Publications included in the analysis.

| Publications | Sampling years | Area | Sample | Infection | Prevalence |
| --- | --- | --- | --- | --- | --- |
| Zheng(Dan et al., 2023) | 2019-2021 | East China Sea | 512 | 182 | 35.55% |
| Ren(Yan-yan et al., 2023) | 2018-2021 | Bohai Sea | 289 | 84 | 29.07% |
| Cai(Wu-wei et al., 2023) | 2021 | East China Sea | 177 | 73 | 41.24% |
| Wei(Zhuo-chao et al., 2020) | 2015-2018 | Bohai Sea | 303 | 158 | 52.15% |
| Zhang(Xue-yan et al., 2020) | 2018 | Yellow Sea | 143 | 84 | 58.74% |
| Lin(Chen-xin et al., 2019) | 2016-2018 | East China Sea | 810 | 277 | 34.20% |
| Li(Ai-hua et al., 2022) | 2017-2020 | Yellow Sea | 388 | 91 | 23.45% |
| Chen(Jun-hua et al., 2014) | 2013 | South China Sea | 382 | 181 | 47.38% |
| Zhang(Jun-he et al., 2010) | 2007-2009 | East China Sea | 444 | 218 | 49.10% |
| Lu(Lu et al., 2018) | 2015-2017 | East China Sea | 684 | 204 | 29.82% |
| Gong(Chun-bo et al., 2018) | 2016-2017 | Bohai Sea | 708 | 112 | 15.82% |
| Li(Xiao-jun et al., 2009) | 2005-2008 | East China Sea | 206 | 134 | 65.05% |
| Ye(Li-ping et al., 2006) | 2004-2005 | East China Sea | 310 | 135 | 43.55% |
| Lin(Chen-xin et al., 2017) | 2012-2016 | East China Sea | 492 | 88 | 17.89% |
| Leng(Shu-zhen, 2009) | 2008 | East China Sea | 399 | 110 | 27.57% |
| Li(Liang et al., 2007) | 2005-2006 | Bohai Sea | 396 | 162 | 40.91% |
| Guan(Bo-yu et al., 2017) | 2011-2016 | South China Sea | 144 | 0 | 0.00% |
| Bao(Min and Kai-shuai, 2012) | 2011 | Bohai Sea | 491 | 182 | 37.07% |
| Xu(Yang et al., 2018) | 2017 | Yellow Sea | 380 | 138 | 36.32% |
| Huang(Guang-ping, 2013) | 2010 | South China Sea | 409 | 226 | 55.26% |
| Ye(Bing et al., 2017) | 2017 | Yellow Sea | 169 | 28 | 16.57% |
| Zhang(Xiao-ping et al., 2012) | 2005-2010 | East China Sea | 433 | 55 | 12.70% |
| Li(Yu-tao et al., 2014) | 2014 | East China Sea | 708 | 517 | 73.02% |
| Zhang(Chun-ling, 2021) | 2019-2020 | Multi-sea areas | 851 | 296 | 34.78% |
| Zhang(Wen-qian et al., 2017) | 2016 | Bohai Sea | 256 | 170 | 66.41% |
| Feng(Long-ji, 2022) | 2022 | Inland | 431 | 99 | 22.97% |
| Cao(Ting-sheng, 2009) | 2007-2008 | East China Sea | 196 | 134 | 68.37% |
| Li(Xiao-jun et al., 2016) | 2008-2010 | East China Sea | 378 | 219 | 57.94% |
| Zhou(Jing-yao et al., 2017) | 2013-2014 | East China Sea | 89 | 82 | 92.13% |
| Chen(Chen et al., 2018) | 2017 | East China Sea | 204 | 204 | 100.00% |
| Li(Li et al., 2017) | 2011 | Yellow Sea | 85 | 85 | 100.00% |
| This study | 2023 | Multi-sea areas | 187 | 79 | 42.25% |

Reference

Ai-hua, L., Lin, W., Sha-sha, L., and Wan-jun, Z. (2022). Prevalence of parasitic infections in fish from markets in Zhenjiang City from 2017 to 2020. *Chinese Journal of Schistosomiasis Control* 34(05)**,** 527-530. doi: 10.16250/j.32.1374.2021178. (In Chinese).

Bing, Y., Zhong-qing, S., Xu-yan, S., Xue-xiang, S., Dan-dan, L., Nan, X., et al. (2017). Research Anisakis infecting in marine fish in Shandong Qingdao. *Journal of Medical Pest Control* 33(09)**,** 985-986. doi: CNKI:SUN:YXDZ.0.2017-09-024. (In Chinese).

Bo-yu, G., Bai-nian, L., Xiu-yun, Z., Rui-mei, Z., Er-ming, W., Jiong-bu, F., et al. (2017). Survey on parasite contamination of food sold in Jiangmen City. *Journal of Diseases Monitor and Control* 11(3)**,** 3. doi: (In Chinese).

Chen-xin, L., Shi-han, L., Wei-wei, C., Shu-ling, H., and Dian-wei, J. (2017). Parasite pollution in aquatic products marketed in Fujian Province. *Chinese Journal of Zoonoses* 33(06)**,** 564-568. doi: 10.3969/j.issn.1002-2694.2017.06.018. (In Chinese).

Chen-xin, L., Shu-ling, H., Shi-han, L., Dian-wei, J., and Han-guo, X. (2019). Infection and species identification of Anisakis larvae in sea fishes along Fujian coast. *Chinese journal of parasitiology and parasitic disease* 37(04)**,** 417-421. doi: 10.12140/j.issn.1000-7423.2019.04.008. (In Chinese).

Chen, H.X., Zhang, L.P., Gibson, D.I., Lü, L., Xu, Z., Li, H.T., et al. (2018). Detection of ascaridoid nematode parasites in the important marine food-fish Conger myriaster (Brevoort) (Anguilliformes: Congridae) from the Zhoushan Fishery, China. *Parasit Vectors* 11(1)**,** 274. doi: 10.1186/s13071-018-2850-4.

Chun-bo, G., Zhao-xia, W., Feng-guang, D., Yu-fang, X., and Yue-lin, S. (2018). Investigation on infection of Anisakis sp. 3^rd^ stage larvae in fresh marine fish for sale in Yantai from 2016 to 2017. *Modern Preventive Medcine* 45(10)**,** 1766-1768. doi: CNKI:SUN:XDYF.0.2018-10-011. (In Chinese).

Chun-ling, Z. (2021). *Investigation of Anisakis infection in coastal fishes of China and establishmen of detection methods.* Jilin University.

Dan, Z., Chen-xin, L., Wu-wei, C., and Han-guo, X. (2023). Investigation of Anisakis spp. infection in marine fish in Fujian Province, 2019-2021. *Chinese Journal of Parasitology and Parasitic Diseases* 41(2)**,** 238-240. doi: 10.12140/j.issn.1000-7423.2023.02.019. (In Chinese).

Guang-ping, H. (2013). *Molecular identification and phylogenetic analysis of Anisakidae larvae from the marine fishes of Perciformes in the South China Sea.* Hebei Normal University.

Jing-yao, Z., Qi, L., Hui, Z., Jun-he, Z., and Zhou-xia, G. (2017). Investigation and molecular identification of Anisakis infection in marine fish from Zhoushan fishery. *China Preventive Medicine Journal* 29(07)**,** 694-697+701. doi: 10.19485/j.cnki.issn1007-0931.2017.07.010. (In Chinese).

Jun-he, Z., Qi, L., Qian-tong, Z., Wei-xian, H., Ke-fengh, L., and Xu, X. (2010). A study of the infection and physicochemical characteristics of the marine fish infected by Anisakis L3 caught in Zhoushan Fishery. *Chinese Journal of Preventive Medicine* 44(11)**,** 6. doi: 10.3760/cma.j.issn.0253-9624.2010.11.018. (In Chinese).

Jun-hua, C., Zhi-xia, X., Guang-xing, X., Jian-yun, H., Hong-hui, C., Shi-zun, S., et al. (2014). Anisakis simplex larvae: Infection status in marine fished for sale in Shantou. *Chinese journal of parasitiology and parasitic disease* 32(03)**,** 212-216. doi: CNKI:SUN:ZJSB.0.2014-03-014. (In Chinese).

Li-ping, Y., Feng, S., Guo-zhang, X., Feng, L., and Zhi-hua, C. (2006). A survey of the infection of fish in the East China Sea with Anisakis larvae. *China Preventive Medicine Journal* 18(07)**,** 31+34. doi: 10.19485/j.cnki.issn1007-0931.2006.07.019. (In Chinese).

Li, L., Zhao, J.Y., Chen, H.X., Ju, H.D., An, M., Xu, Z., et al. (2017). Survey for the presence of ascaridoid larvae in the cinnamon flounder Pseudorhombus cinnamoneus (Temminck & Schlegel) (Pleuronectiformes: Paralichthyidae). *Int J Food Microbiol* 241**,** 108-116. doi: 10.1016/j.ijfoodmicro.2016.10.018.

Liang, L., Zhen, X., and Lu-ping, Z. (2007). Investigation on the nematode of *Hysterothylacium aduncum* (Anisakisdae) from Bohai Sea and Yellow Sea in China. *Chinese journal of parasitiology and parasitic disease* 25(05)**,** 364-367. doi: 10.3969/j.issn.1000-7423.2007.05.002. (In Chinese).

Long-ji, F. (2022). *Species identification and infection analysis of parasitic nematodes of Schizothorax from Yarkand river basin.* Xinjiang Agricultural University.

Lu, L., Shou-fu, J., Yan-yan, H., Xiao-ping, Z., Xiao-jiang, M., Ying-jun, H., et al. (2018). Investigation on the animal foods contaminated with parasites in markets in Huangpu Distric of Shanghai from 2015 to 2017. *Journal of Tropical Diseases and Parasitology* 16(1)**,** 3. doi: 10.3969/j.issn.1672—2302.2018.01.007. (In Chinese).

Min, B., and Kai-shuai, S. (2012). Anisakidae infection investigation of commercial marine fish in Jinzhou. *Chinese Journal of Zoonoses* 28(05)**,** 513-514+516. doi: (In Chinese).

Shu-zhen, L. (2009). *Studies on the Identification Method of Anisakid Nematodes in Sea Fish and Investigates on the Infection of Fujian Coast.* Fujian Agriculture and Forestry University.

Ting-sheng, C. (2009). *Study on the detection method of the fish Anisakis.* Zhejiang University.

Wen-qian, Z., Xiao-tong, R., Yu-qi, Z., Xin-xin, G., Yu-mei, Z., and Xue-lian, B. (2017). Investigation of Aniksakis spp. larva infection in marine fish for sale in Yantai City. *Chinese journal of parasitiology and parasitic disease* 35(05)**,** 472-477. doi: CNKI:SUN:ZJSB.0.2017-05-013. (In Chinese).

Wu-wei, C., Chen-xin, L., Dan, Z., and Han-guo, X. (2023). Prevalence of Anisakis infections in marine fishes in Eastern Fujian Fishing Groud of Fujian Province. *Chinese Journal of Schistosomiasis Control* 35(01)**,** 78-81. doi: 10.16250/j.32.1374.2022119. (In Chinese).

Xiao-jun, L., Ting-sheng, C., Ai-fang, D., and Wei-ming, C. (2009). Preliminary survey of Anisakis larvae infections in marine fish from the East China Sea. *Chinese Journal of Veterinary Medicine* 45(10)**,** 76-77. doi: CNKI:SUN:ZSYZ.0.2009-10-035. (In Chinese).

Xiao-jun, L., Yuan-yuan, S., jie, B., Lu-min, C., yuan, Z., Chang-lin, S., et al. (2016). Survey on the larval infection of Anisakis in marine fish entering and leaving the port of Zhoushan. *Animal Husbandry and Veterinary Medicine* 48(05)**,** 119-122. doi: CNKI:SUN:XMYS.0.2016-05-028. (In Chinese).

Xiao-ping, Z., Shou-fu, J., Guo-bao, H., Ying-hua, F., Yan-yan, H., Xiao-jiang, M., et al. (2012). Investigation on food contamination with parasites in Shanghai market. *Chinese Journal of Schistosomiasis Control* 24(4)**,** 6. doi: 10.16250/j.32.1374.2012.04.006. (In Chinese).

Xue-yan, Z., Min, Y., Qing-qing, Z., Ying, W., and Bo-chao, S. (2020). Investigation of Anisakis infections in market⁃available marine fish in Dongtai City. *Chinese Journal of Schistosomiasis Control* 32(04)**,** 426-427+440. doi: 10.16250/j.32.1374.2019267. (In Chinese).

Yan-yan, R., Bo, D., Nan-nan, C., Min, X., and Wen-xin, L. (2023). Investigation and molecular identification of Anisakis infection in marine fish sold in Fuxin, Liaoning Province. *China Tropical Medicine* 23(05)**,** 489-494. doi: 10.13604/j.cnki.46-1064/r.2023.05.09. (In Chinese).

Yang, X., Qiao-ying, Z., Zhi-hua, S., Li-Xiao, Z., Nan, W., Xiao-lei, L., et al. (2018). Preliminary investigation of Anisakis infection in commercial marine fish in Lianyungang City. *Chinese Journal of Veterinary Science* 38(12)**,** 2343-2347. doi: 10.16303/j.cnki.1005-4545.2018.12.20. (In Chinese).

Yu-tao, L., Wei-jiang, Y., Chao-chao, L., Hu-yan, H., Si-hui, Z., and Xin, Q. (2014). A Survey on infection of Anisakis in export seafood from Taizhou. *Tianjin Agricultural Sciences* 20(05)**,** 74-76. doi: 10.3969/j.issn.1006-6500.2014.5.016. (In Chinese).

Zhuo-chao, W., Wei, Z., and Xiao-li, D. (2020). Investigation of Anisakis larvae infection in marine fish in markets of Anshan City, Liaoning Province. *Chinese Journal of Parasitology and Parasitic Diseases* 38(06)**,** 789-791. doi: 10.12140/j.issn.1000-7423.2020.06.020. (In Chinese).
